# Fish Assemblages Distinguish Eco-Friendly from Conventional Rice Paddies Through Abundance and Biomass Rather than Diversity

**DOI:** 10.3390/biology15161411

**Published:** 2026-08-17

**Authors:** Byung-Mo Lee, Myung-Hyun Kim, Soon-kun Choi, Jinu Eo, Sang-Min Jun

**Affiliations:** 1 Climate Change Assessment Division, National Institute of Agricultural Sciences, Rural Development Administration, Wanju 55365, Republic of Korea; leebm@korea.kr (B.-M.L.); soonkun@korea.kr (S.-k.C.); eojiny@korea.kr (J.E.); luckyjsm@korea.kr (S.-M.J.); 2 Apiculture Division, National Institute of Agricultural Sciences, Rural Development Administration, Wanju 55365, Republic of Korea

**Keywords:** aquatic biodiversity, bioindicators, catch per unit effort, community composition, eco-friendly rice farming, fish assemblages, species-specific response, South Korea

## Abstract

Although rice paddies are agricultural land, they also serve as artificial wetlands that deliver essential ecosystem services. Yet how farming systems affect their fish communities—and which metrics best capture those effects—remains poorly quantified in Asia. We compared fish assemblages between eco-friendly and conventional rice paddies in South Korea across the 2015 rice growing season (May–September), sampling 94 events by fyke net in Dangjin City (2704 individuals; 13 species) and analyzing the data with generalized linear mixed models and multivariate methods. Eco-friendly paddies supported 2.3-fold higher abundance and 2.6-fold higher biomass; differences were negligible in May (Cohen’s *d* ≈ 0) but large from June onward (*d* > 1.0), with a moderate-to-large pooled effect (*d* = 0.76–0.89). Community composition differed significantly (PERMANOVA), and eco-friendly assemblages were more consistent among fields (PERMDISP). *Carassius auratus* and *Misgurnus anguillicaudatus* were 10.8- and 2.7-fold more abundant under eco-friendly management, whereas α-diversity indices (richness, Shannon, Simpson, evenness) showed no differences, as three taxa comprised over 90% of the catch. In these species-poor, dominance-skewed systems, abundance-based and species-specific metrics detected management effects more sensitively than α-diversity indices, complementing rather than replacing them. Because these results derive from a single growing season in one region, however, they should be regarded as specific to comparable systems and require multi-year, multi-region validation before their use in biomonitoring and eco-friendly agricultural policy can be generalized.

## Simple Summary

Rice paddies are not only farmland but also artificial wetlands that shelter aquatic animals such as fish. We asked whether eco-friendly rice farming, which reduces or eliminates synthetic pesticides and fertilizers, supports richer fish life than conventional farming, and which biological measures best reveal such differences. Over one rice-growing season in a single region of South Korea, we sampled fish in eco-friendly and conventional paddies. Eco-friendly paddies held more than twice as many fish and greater fish biomass, and a more consistent community, even though common diversity indices showed no difference. Within this species-poor system, counts of fish were more informative than diversity indices, a finding that should be confirmed across more years and regions.

## 1. Introduction

Rice paddies constitute one of the world’s most extensive agricultural ecosystems, covering approximately 168.4 million hectares globally [1], and they function as surrogate freshwater habitats in many regions where natural wetlands have been lost [2,3]. Beyond producing a staple food for much of humanity, they deliver a broad suite of ecosystem services—including biodiversity conservation, water regulation, and habitat provisioning for aquatic organisms [4]—and agricultural wetlands are increasingly recognized as providing consistently high ecosystem-service value worldwide [5]. This role is especially pronounced in Asia, which produces roughly 90% of the world’s rice and where paddies act as expansive artificial wetlands that sustain diverse aquatic communities. Among these, fish occupy a pivotal position: they contribute to nutrient cycling and food-web maintenance, serve as principal prey for waterbirds and other higher-order predators, and integrate environmental conditions across space and time [6,7,8]. Although rice is predominantly cultivated in tropical and subtropical Asia, extensive temperate rice production also occurs in East Asia.

Since the 1960s, the intensification of rice farming—propelled by the widespread use of synthetic pesticides and chemical fertilizers—has raised sustained concerns about its consequences for aquatic biodiversity and freshwater ecosystem integrity [9]. Pesticides can be directly toxic to non-target aquatic organisms, including fish, whereas nutrient loading from chemical fertilizers promotes eutrophication and habitat degradation in paddy-associated waters [10], and adverse effects of insecticide and fungicide use on farmland biodiversity have been documented across multiple trophic levels [11]. In response, eco-friendly farming—here encompassing organic and pesticide-free systems that minimize or eliminate chemical inputs—has been widely promoted as a means of reconciling production with ecological sustainability [12]. Determining whether such practices deliver measurable ecological benefits, however, requires biological indicators and quantitative metrics that can reliably detect management-driven change and provide an evidence base for agricultural policy.

Fish assemblages are strong candidate indicators in agricultural wetlands because they are sensitive to environmental changes, possess diverse life-history strategies, and occupy diverse positions in food webs [13,14]. Unlike short-lived invertebrates, fish communities integrate environmental conditions over longer temporal and spatial scales, making them well-suited to detecting chronic agricultural impacts [15]. The metrics used to summarize such assemblages, however, are decisive. Traditional α-diversity indices—species richness, Shannon, Simpson, and evenness—implicitly assume assemblages rich enough for compositional turnover to register. In species-poor, dominance-skewed systems such as temperate rice paddies, where a few tolerant taxa numerically dominate [16,17], these indices may remain insensitive to management effects even when populations respond strongly. Consistent with these findings, meta-analyses of organic farming report that biodiversity recovers mainly through increases in organism abundance rather than through large gains in species richness [12,18]. Abundance-based and species-specific metrics may therefore capture management signals that α-diversity indices overlook—an expectation seldom-tested for fish in rice paddies. Contrasts between abundance-based and diversity-based responses have previously been noted in studies of agricultural biodiversity, aquatic macroinvertebrates, and organic farming; the present study extends this line of work to paddy fish assemblages under contrasting management rather than addressing a wholly overlooked question.

Farming practices are known to shape aquatic communities in rice paddies, yet the evidence remains uneven. Most studies have targeted aquatic invertebrates, which frequently respond positively to organic management [19,20,21,22], although responses can differ markedly even among invertebrate groups [23]. In Korea, nationwide surveys reported higher benthic-invertebrate diversity and abundance in organic than in conventional paddies [24], whereas comparisons involving vertebrates are scarce and often address only a single species or a single phase of cultivation—for example, prey availability for great egrets during the transplanting season [25]—rather than whole fish communities across the growing season. Elsewhere in East Asia, a large-scale Japanese study found that organic farming benefited multiple taxa, including loaches (Cobitidae), but that loach abundance was governed more by hydrological management than by farming-system category per se [26], and vertebrate responses have proven more context-dependent than those of invertebrates [27]. Because paddies are periodically inundated and drained, community assembly is further filtered by seasonal hydrology, so that observations at a single time point may misrepresent system differences. Comprehensive, season-long assessments of paddy fish communities—resolving both which taxa colonize and how their populations respond to management—therefore remain a notable gap.

South Korea provides a well-suited setting for addressing this gap. National policies promoting sustainable agriculture have expanded certified eco-friendly rice paddies to approximately 36,837 ha in 2025 (20,691 ha organic and 16,146 ha pesticide-free) [28], corresponding to 4.9% of the country’s 755,952 ha of rice paddies [29]. This policy-driven mosaic of eco-friendly and conventional fields, embedded within intensive production systems, offers a natural contrast for quantifying the ecological effects of farming practice. Fish are a particularly informative target in this context, both because they are primary prey for waterbirds and other predators and because quantitative data on paddy fish communities—and on whether, and through which metrics, they can distinguish farming systems—remain limited.

Accordingly, we compared fish assemblages between eco-friendly and conventional rice paddies in South Korea across an entire growing season (May–September). Our objectives were to (1) quantify differences in fish abundance, biomass, and community composition between the two systems and characterize their seasonal dynamics; (2) identify species-specific and indicator-species responses to farming practice; and (3) compare the sensitivity of abundance-based metrics with that of α-diversity indices for detecting management effects in this species-poor assemblage. Because reliable quantification of abundance and biomass still depends on standardized, effort-based capture rather than emerging molecular surveys [30], we paired fyke-net sampling with population- and community-level analyses. In doing so, the study aims to clarify which biomonitoring metrics best capture the ecological outcomes of eco-friendly farming and to provide an evidence base for sustainable rice-paddy management across Asian agricultural landscapes.

## 2. Materials and Methods

### 2.1. Study Area

**Figure 1:**
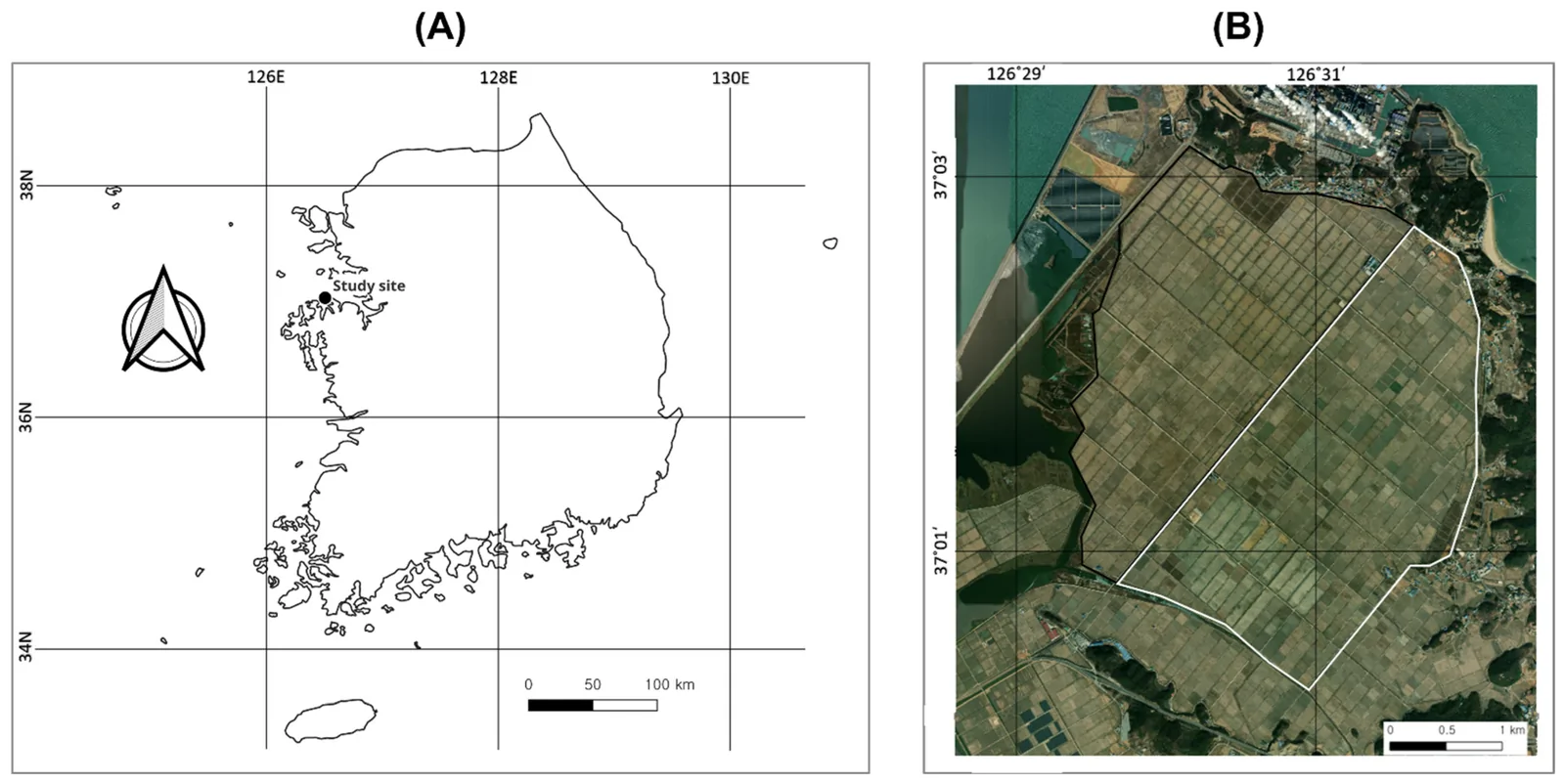
Location of the study site. (**A**) Map of South Korea showing the location of the study site (black dot); (**B**) high-resolution satellite image of the study site (black boundary: eco-friendly paddies; white boundary: conventional paddies).

The study was conducted in rice paddies in Seokmun-myeon, Dangjin-si, Chungcheongnam-do, South Korea (37°02′ N, 126°30′ E) (Figure 1), a temperate region, during the 2015 rice cultivation period (May–September). The rice paddies in this region were developed on 3904 ha of reclaimed land starting in 1979 as part of the Daeho Large-scale Agricultural Development Project. Initially, all rice paddies were managed using conventional methods, but since 1999, eco-friendly cultivation practices have been gradually introduced, and by 2015, approximately 574.2 ha were managed as eco-friendly rice paddies. The eco-friendly and conventional paddies are adjacent to each other, separated by farm roads, levees, and water channels, and irrigated by the same drainage system. These characteristics make the region highly suitable for comparative studies of the two farming systems, providing a natural control for environmental variation and minimizing confounding landscape-level effects.

Cultivation methods in the study paddies were classified as “eco-friendly” (hereafter EF) or “conventional” (hereafter CF) based on pesticide and chemical fertilizer management practices. Eco-friendly paddies were managed with minimal to no synthetic pesticide or chemical fertilizer applications and were operated under a contract-farming system supervised by the Korea Rural Community Corporation. Most eco-friendly paddies were completely pesticide-free, whereas some were managed under a low-pesticide regime, for which occasional limited pesticide applications were recorded. At the time of the survey (2015), the low-pesticide category was included within the national eco-friendly agricultural certification; this category was abolished entirely on 1 January 2016. Conventional paddies were privately managed by individual farmers following standard input-intensive rice cultivation, including the use of chemical fertilizers, insecticides such as clothianidin, fungicides such as tiadinil, and herbicides such as pyrazosulfuron–ethyl and fentrazamide, applied according to local practices. The two systems were thus clearly distinguished by their management regime and farming intensity. Nonetheless, because field-level quantitative records of pesticide and fertilizer application were not available, mechanistic attributions to agrochemical reduction are treated as hypotheses (Section 4).

### 2.2. Fish Sampling and Identification

We employed a repeated cross-sectional sampling design in which 10 eco-friendly and 10 conventional paddies were independently selected each month across five sampling periods (May, June, July, August, September 2015), resulting in 100 planned sampling events (10 paddies × 2 farming systems × 5 months). Because individual paddies could not always be revisited throughout the growing season, paddies were selected each month from the broader pool of eco-friendly and conventional fields within the study area and were not necessarily the same paddies across months. Consequently, each monthly sampling event was treated as an independent observational unit, allowing seasonal comparisons while avoiding the bias that would arise from treating these as incomplete repeated measurements of the same fields. Due to occasional trap loss and disturbance by animals such as raccoon dogs, reliable data were obtained from 94 sampling events (48 from eco-friendly paddies, 46 from conventional paddies). Six sampling events (two from eco-friendly paddies, four from conventional paddies) were excluded due to complete trap loss or insufficient functional traps (<2 traps per paddy).

Fish sampling followed standardized protocols using fyke nets as passive sampling gear. Three cylindrical fyke nets (28 cm long, 13 cm diameter, 4 mm mesh) were deployed in each paddy at minimum intervals of 5 m and retrieved after 24 h. Nets were positioned with entrances fully submerged, with slight excavation when necessary in shallow areas. Each fyke net was equipped with a luminescent ring at the entrance to attract fish with light, and 6 g of commercial fish meal was placed inside as bait. Sampling was conducted on five dates: 7–8 May, 7–8 June, 1–2 July, 6–7 August, and 2–3 September 2015. All captured fish were preserved in 10% formalin and transported to the laboratory for identification using standard Korean fish identification keys [31,32].

### 2.3. Data Analysis

#### 2.3.1. Quantification of Community Attributes

Each month–paddy observation was treated as a sampling unit for analysis. Abundance was assessed as the total number of individuals captured, expressed as individual-based catch per unit effort (CPUE_ind, individuals trap^−1^), which was calculated by dividing the total number of individuals by the number of functional traps deployed during each monthly sampling event. Species-specific abundances were recorded for all identified taxa. Biomass per unit effort (CPUE_bio) was quantified as two distinct metrics: wet weight (CPUE_bio_wet, g trap^−1^) and dry weight (CPUE_bio_dry, g trap^−1^). Wet weight represented the fresh body mass at capture, while dry weight was determined after drying individuals to a constant mass. For each sampling event, these values were calculated by summing the respective biomass of all individuals and dividing by the number of functional traps.

Species-specific preferences were quantified as log_10_ abundance ratios. To include all identified taxa and avoid undefined values from zero counts, a constant of 1 was added to the total abundance of each species in both systems: log_10_[(N_eco_ + 1)/(N_conv_ + 1)]. Higher positive values indicated a stronger association with eco-friendly management, while negative values indicated an association with conventional management.

Diversity was characterized by calculating species richness (*S*), Shannon–Wiener diversity index (*H’* = −Σp_i_lnp_i_), Simpson’s dominance index (*D* = Σp_i_^2^), and Pielou’s evenness (*J’* = H’/ln*S*). Sampling events with no captured individuals were excluded from diversity calculations, and events with only a single species were excluded from evenness analysis.

#### 2.3.2. Statistical Analysis

All statistical analyses were conducted in R version 4.6.1 [33], with significance set at α = 0.05. Because the abundance and biomass data were right-skewed, overdispersed, and zero-inflated, and because sampling events from spatially adjacent fields shared the same irrigation and drainage network, between-system differences were modelled using generalized linear mixed models (GLMMs) rather than independent two-sample tests. Individual abundance (CPUE_ind) was modelled with a negative binomial error structure, and wet and dry biomass (CPUE_bio_wet, CPUE_bio_dry) with a Tweedie (log-link) structure, using the glmmTMB package [34]. Models included farming system and sampling month as fixed effects and a random intercept for field identity to account for potential non-independence among observations collected within the same field, as well as spatial clustering among neighboring paddies connected by the same irrigation and drainage network. In all models, the natural logarithm of the number of functional traps per event was included as an offset, so that between-system contrasts are expressed on a per-trap (catch-per-unit-effort) basis. Model assumptions were evaluated using simulation-based residual diagnostics implemented in the DHARMa package [35], including inspection of residual distributions, tests for overdispersion, and assessment of zero inflation. Between-system contrasts were expressed as model-based rate ratios with 95% confidence intervals (estimated with the emmeans package [36]), and the farming system × month interaction was used to test whether the magnitude of between-system differences varied seasonally. For descriptive comparison, standardized effect sizes were additionally computed on catch-per-unit-effort values, both overall and by month, as Cohen’s *d* [37] and the rank-biserial correlation, alongside the model-based estimates. Although raw counts contained many zeros, zero inflation was tested rather than assumed: because these DHARMa diagnostics indicated no residual overdispersion or zero inflation, zero-inflated and hurdle alternatives were rejected in favour of the more parsimonious negative binomial (abundance) and Tweedie (biomass) structures (Figure S1). This choice was corroborated by Akaike Information Criterion (AIC) comparison, in which the negative binomial had the lowest AIC and adding zero-inflation or hurdle components did not improve fit, while Tweedie clearly outperformed a Gaussian alternative for biomass (Table S4). The annotated model-selection code is provided in the Supplementary Materials. Because the specific paddies sampled were not fully identical across months, the design is not a strict repeated-measures design; the field-level random intercept accounts for within-field non-independence and spatial clustering among hydrologically connected paddies but does not explicitly model temporal autocorrelation, which is acknowledged as a limitation.

Species-specific responses to farming system were evaluated for taxa occurring in at least 5% of sampling events using the same GLMM framework, and *p*-values across species were adjusted for multiple comparisons using the Benjamini–Hochberg false discovery rate (FDR) procedure [38]. Species-level associations with the two systems were additionally summarized as log_10_ abundance ratios, calculated as log_10_[(N_EF + 1)/(N_CF + 1)], where positive values indicate affinity for eco-friendly paddies.

Differences in community composition were tested at the level of individual sampling events using permutational multivariate analysis of variance (PERMANOVA [39]; adonis2 in the vegan package [40], 9999 permutations) on a Bray–Curtis dissimilarity matrix of event-level species catch-per-unit-effort (two events with no fish were excluded, as the Bray–Curtis measure is undefined for empty samples), with farming system and month as factors. Homogeneity of multivariate dispersion among systems was evaluated using PERMDISP (betadisper) to determine whether significant PERMANOVA results reflected differences in centroid location rather than differences in dispersion. Community structure was visualized with non-metric multidimensional scaling (NMDS) applied to square-root-transformed CPUE, which was used to reduce the influence of highly abundant species before calculating Bray–Curtis dissimilarities. To formally identify taxa characteristic of each farming system, indicator species analysis was performed using the group-equalized IndVal.g index (multipatt in the indicspecies package [41]) with 9999 permutations, reporting each species’ specificity (component A), fidelity (component B), and Benjamini–Hochberg FDR-adjusted significance. α-diversity indices (species richness S, Shannon–Wiener H’, Simpson’s D, and Pielou’s J’) were compared between farming systems using the GLMM framework described above. In addition, because the small within-month sample sizes (8–10 events per system) precluded stable mixed-model fitting, monthly differences in each α-diversity index between the two systems were assessed separately by month using two-sided Mann–Whitney U tests. Figures were produced with the ggplot2 package [42].

## 3. Results

### 3.1. General Characteristics of Fish Assemblage

A total of 2704 individual fish representing 13 species from 6 families were collected during the study (Table 1). The fish assemblage was taxonomically dominated by Cyprinidae, which comprised 5 species (38.5% of the total species richness), followed by Gobiidae with 3 species (23.1%) and Cobitidae with 2 species (15.4%). Overall, the assemblage consisted primarily of small-bodied freshwater fishes commonly associated with shallow agricultural wetlands.

Eco-friendly paddies yielded 1888 individuals (69.8% of total catch), whereas conventional paddies yielded 816 individuals (30.2%). *Misgurnus anguillicaudatus* was the most abundant species in both farming systems, accounting for 68.8% and 59.3% of individuals in eco-friendly and conventional paddies, respectively. Across all samples, *M. anguillicaudatus* represented 65.9% of total catch, followed by *Aphyocypris chinensis* (15.8%) and *Carassius auratus* (12.4%), while the remaining species each accounted for less than 3% of total abundance (Table 1).

### 3.2. Differences in Abundance and Biomass

**Figure 2:**
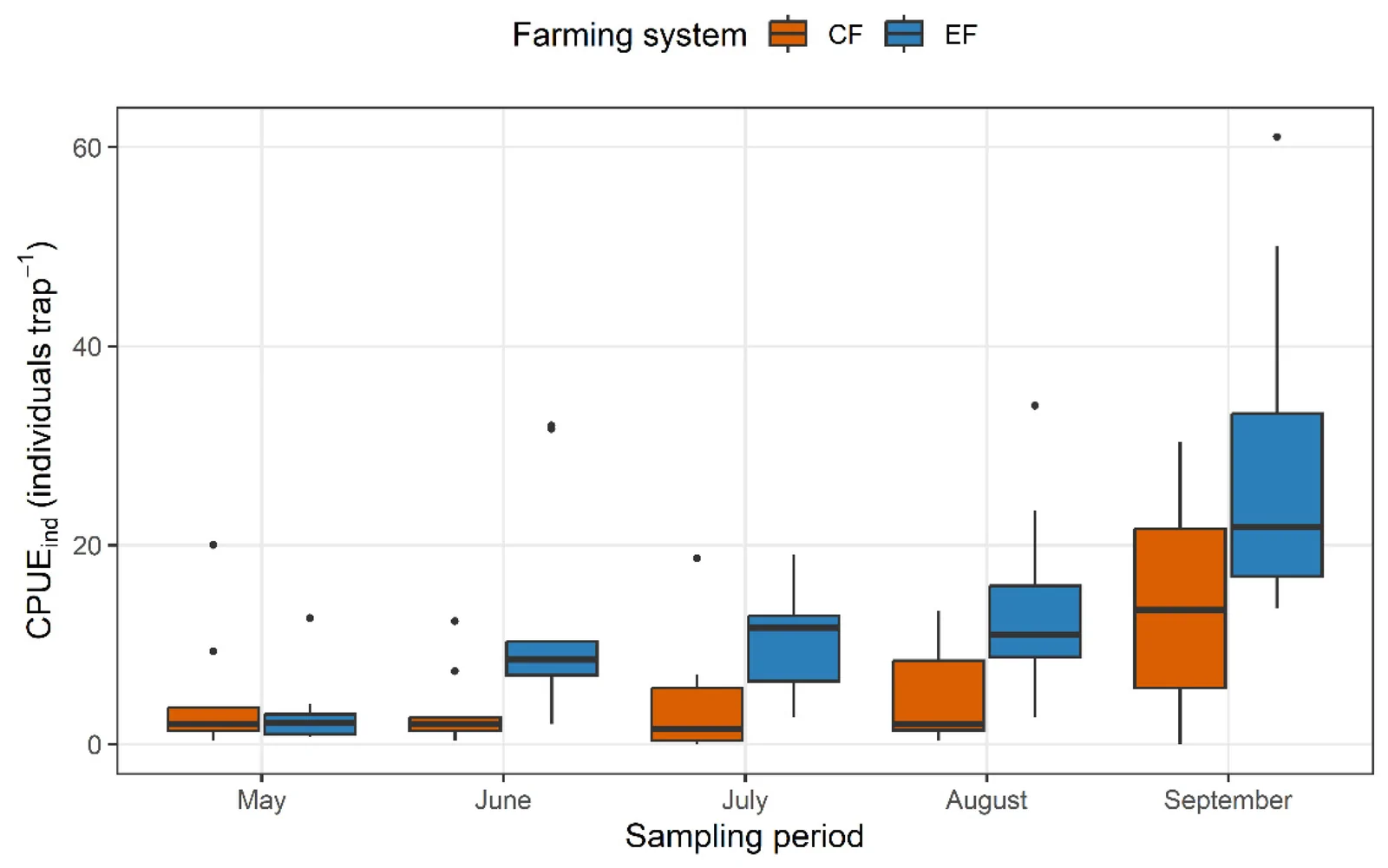
Monthly individual-based catch per unit effort (CPUE ind; individuals trap^−1^) of fish in eco-friendly (EF) and conventional (CF) rice paddies during the 2015 growing season (May–September). Boxes show the median and interquartile range (IQR); whiskers extend to 1.5 × IQR.

CPUE showed consistent and significant differences between farming systems across most of the cultivation period. Overall abundance (CPUE_ind) was significantly higher in eco-friendly paddies (14.0 ± 12.7 individuals trap^−1^) compared to conventional paddies (6.1 ± 7.5 individuals trap^−1^; Mann–Whitney U test, *p* < 0.001, Cohen’s *d* = 0.76; Table 2 and Figure 2), representing a moderate-to-large overall effect. A negative binomial GLMM that accounted for sampling effort (number of functional traps) and the repeated sampling of the same fields (random intercept for field) confirmed this difference (rate ratio EF/CF = 2.39, 95% CI 1.48–3.87, *p* < 0.001) and revealed a significant farming system × month interaction (*p* = 0.039): the difference was negligible in May (rate ratio 0.77, *p* = 0.557) but pronounced from June to August (rate ratios 3.1–3.9, all *p* < 0.01) and weaker in September (rate ratio 1.90, *p* = 0.114). Model diagnostics (simulation-based quantile residuals) indicated no overdispersion or zero-inflation and uniform residuals.

**Figure 3:**
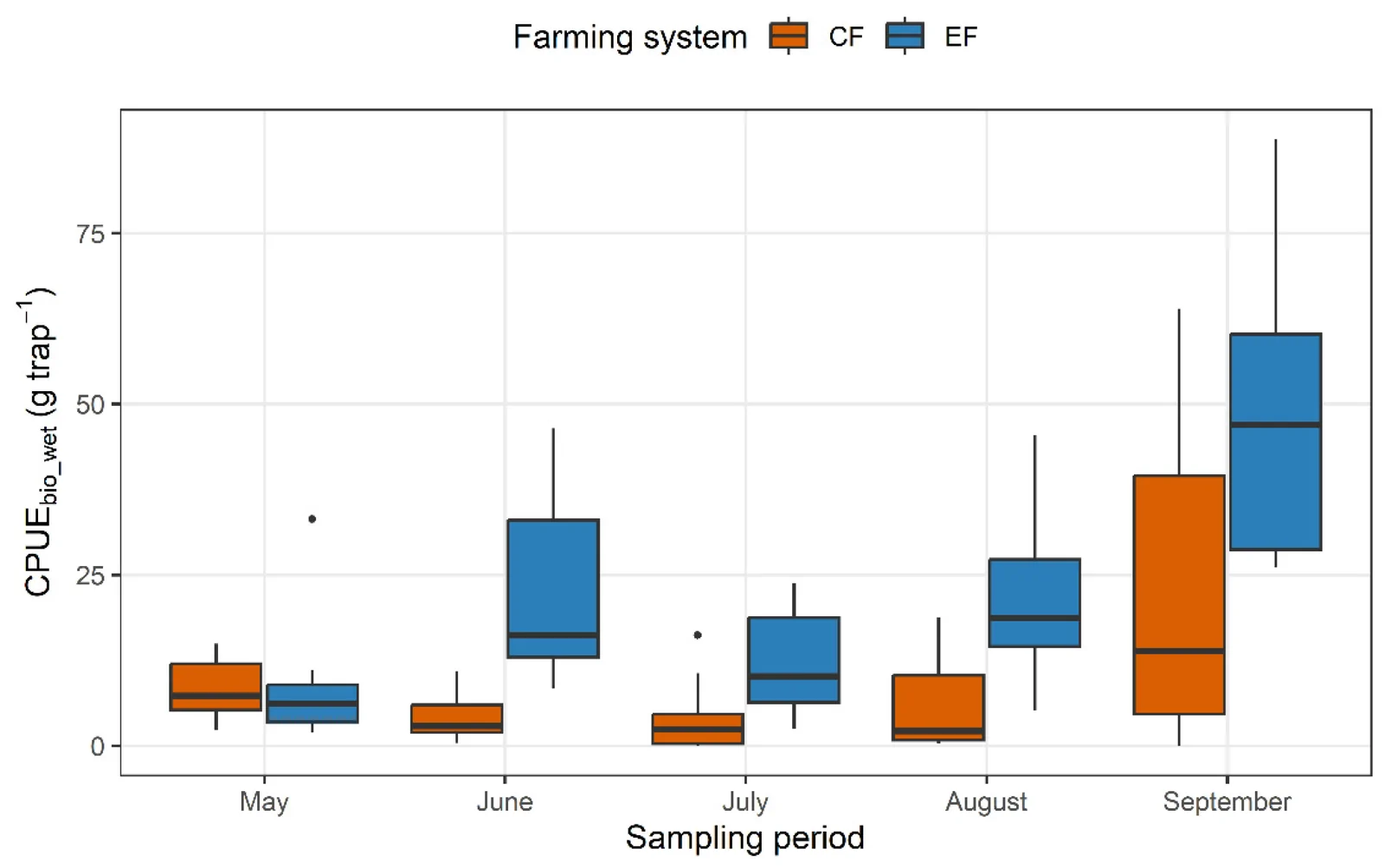
Monthly wet-biomass catch per unit effort (CPUE bio_wet; g trap^−1^) of fish in eco-friendly (EF) and conventional (CF) rice paddies during the 2015 growing season (May–September). Boxes show the median and interquartile range (IQR); whiskers extend to 1.5 × IQR.

Biomass indicators exhibited even more pronounced differences (Table 3 and Table S3, Figure 3). Wet weight biomass (CPUE_bio_wet) was 2.6-fold higher in eco-friendly paddies (23.4 ± 19.7 g/trap) compared to conventional ones (8.9 ± 13.1 g trap^−1^; *p* < 0.001, Cohen’s *d* = 0.86). Similarly, dry weight biomass (CPUE_bio_dry) showed a 2.5-fold difference, with eco-friendly paddies yielding 4.4 ± 3.4 g trap^−1^ versus 1.7 ± 2.4 g trap^−1^ in conventional paddies (*p* < 0.001, Cohen’s *d* = 0.89). Both abundance and biomass were thus consistently higher under eco-friendly management. Tweedie GLMMs with the same offset and random-effect structure confirmed these biomass differences (wet: rate ratio EF/CF = 2.62, 95% CI 1.82–3.76, *p* < 0.001; dry: rate ratio = 2.45, 95% CI 1.73–3.45, *p* < 0.001), with a farming system × month interaction that was significant for dry biomass (*p* = 0.04) and marginal for wet biomass (*p* = 0.05).

Temporally, effect sizes revealed a clear seasonal pattern (Table 2, Table 3 and Table S3). In May, differences between farming systems were negligible across all metrics (Cohen’s *d* = −0.24 to 0.17, *p* > 0.05). A sharp divergence emerged from June onwards, with effect sizes exceeding *d* = 1.0 for all abundance and biomass metrics throughout June–September (CPUE_ind: *d* = 1.06–1.23; CPUE_bio_wet: *d* = 1.15–1.78; CPUE_bio_dry: *d* = 1.11–1.68), consistent with Cohen’s classification of large effects. The largest between-system difference was observed in June for biomass (Cohen’s *d* = 1.78 for wet weight; 1.68 for dry weight). Although the difference in abundance in September did not reach statistical significance (*p* = 0.114), the effect size remained large (*d* = 1.06), and biomass differences remained significant (*p* < 0.05) through the end of the cultivation period. The monthly distributions of individual abundance and wet biomass are shown in Figure 2 and Figure 3, respectively: The two systems overlapped in May but diverged progressively from June onward, with consistently higher values in eco-friendly paddies.

### 3.3. Species-Specific Responses

**Figure 4:**
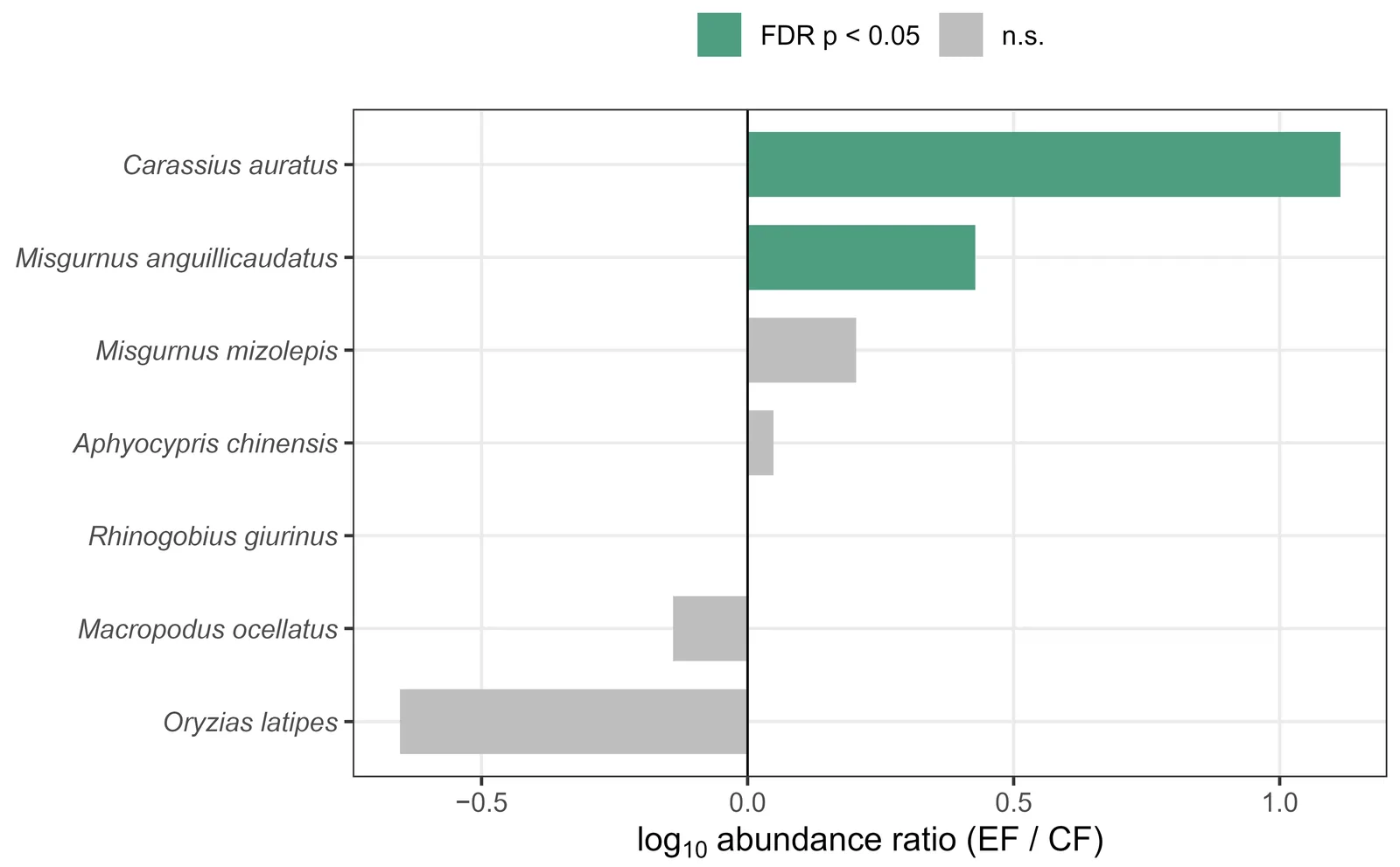
Species-specific preferences for farming systems, showing significant contrasts in both abundance and occurrence between farming systems. Values represent log_10_ abundance ratios between eco-friendly and conventional paddies.

Species exhibited contrasting responses to farming systems (Table S1, Figure 4). The log_10_ abundance ratios visualize the direction and magnitude of species-specific preferences; positive values indicate affinity for eco-friendly paddies, while negative values indicate affinity for conventional paddies.

Of the 13 species recorded, only two showed statistically significant differences between farming systems. Species-level negative binomial GLMMs (field as a random intercept, offset for trap number) with Benjamini–Hochberg FDR correction confirmed both effects: *M. anguillicaudatus* was more abundant in eco-friendly paddies (rate ratio = 2.67, FDR-adjusted *p* < 0.001) and *C. auratus* markedly so (rate ratio = 15.5, FDR-adjusted *p* = 0.004), whereas no other species, including the conventional-leaning *O. latipes* (rate ratio = 0.27, FDR-adjusted *p* = 0.702), differed significantly. *M. anguillicaudatus*, the numerically dominant species, was 2.7 times more abundant in eco-friendly paddies (CPUE_ind.: 9.58 ± 8.63 vs. 3.57 ± 5.50 individuals trap^−1^, *p* < 0.001; Log_10_ ratio = 0.43). The most pronounced preference for eco-friendly paddies was observed in *C. auratus* (Log_10_ ratio = 1.11), which showed a 10.8-fold higher abundance in eco-friendly paddies (CPUE_ind.: 2.38 ± 6.30 vs. 0.22 ± 1.12 individuals trap^−1^, *p* = 0.004). This species was detected in 45.8% of eco-friendly sampling events, compared to only 10.9% of conventional events.

The remaining 11 species showed no statistically significant differences between farming systems (*p* > 0.05 for all; Table S1). While *O. latipes* showed a numerical tendency toward conventional paddies (Log_10_ ratio = −0.65; CPUE_ind.: 0.11 ± 0.65 vs. 0.38 ± 1.70 individuals trap^−1^), this difference was not statistically significant (*p* = 0.702). Notably, *T. bifasciatus* and *T. brevispinis* were absent from all eco-friendly paddies and detected only in conventional paddies, though their overall abundance was too low to reach statistical significance. No species exhibited a statistically significant preference for conventional paddies.

### 3.4. Community Diversity and Composition

Traditional α-diversity indices showed no significant differences between eco-friendly and conventional paddies (Table 4; generalized linear mixed models, *p* > 0.05 for all metrics). Mean Shannon–Wiener diversity (*H’*) was 0.47 ± 0.39 in eco-friendly paddies and 0.39 ± 0.43 in conventional paddies (*p* = 0.358). Similarly, Simpson’s dominance index (*D*) was 0.73 ± 0.23 and 0.77 ± 0.24, respectively (*p* = 0.422). Pielou’s evenness (*J’*) and species richness (*S*) also exhibited no significant disparities between the two farming systems (*p* = 0.267 and 0.206, respectively).

Monthly patterns of diversity (Table S2) remained consistent throughout the study period, with no systematic differences observed in any index from May to September (*p* > 0.10 in all cases).

**Figure 5:**
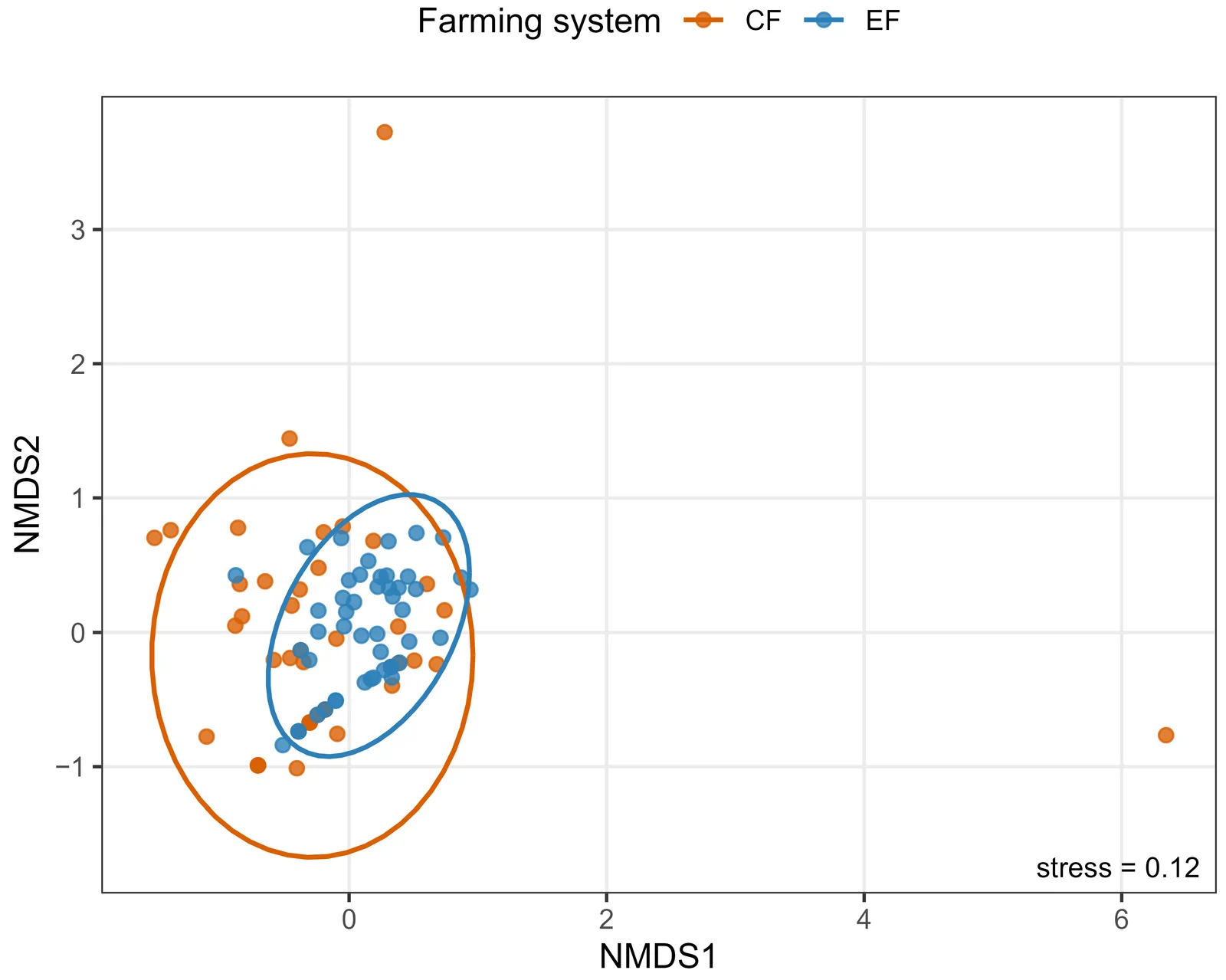
Non-metric multidimensional scaling (NMDS) ordination of fish assemblage composition, based on Bray–Curtis dissimilarities of square-root-transformed CPUE. Points are sampling events; ellipses are 95% confidence ellipses for each farming system (stress = 0.12).

Despite the similarities in these traditional α-diversity metrics, event-level community composition differed significantly between farming systems (PERMANOVA on Bray–Curtis dissimilarities of catch-per-trap data: pseudo-F (1, 86) = 9.56, R^2^ = 0.086, *p* < 0.001, 9999 permutations; two events with no fish were excluded; sampling month was also significant, R^2^ = 0.137, *p* < 0.001). Multivariate dispersion likewise differed between systems (PERMDISP: F = 12.09, *p* = 0.001), with conventional paddies showing greater among-event heterogeneity than eco-friendly paddies (mean distance to group centroid 0.52 vs. 0.40), a pattern also visible as a tighter clustering of eco-friendly events in the NMDS ordination (stress = 0.12; Figure 5). Because dispersion also differed between systems, the PERMANOVA result is interpreted with caution, as it may partly reflect this difference in dispersion in addition to a shift in assemblage centroid; at the same time, the greater homogeneity of eco-friendly assemblages is itself an interpretable ecological signal. Thus, eco-friendly paddies supported an assemblage that was both compositionally distinct and more consistent among events. Indicator species analysis identified *C. auratus* as the only significant indicator species, characterizing eco-friendly paddies (IndVal = 0.65, FDR-adjusted *p* < 0.01); *M. anguillicaudatus*, although the most abundant taxon, occurred in almost all events of both systems and was therefore not system-specific. Community-level composition thus differed between systems even where α-diversity indices did not.

## 4. Discussion

### 4.1. Fish Communities as Ecological Indicators

Eco-friendly paddies supported 2.3-fold higher fish abundance and 2.6-fold greater biomass than conventional paddies, providing clear biological evidence that farming practices strongly influence aquatic communities in rice paddy ecosystems. Because fish integrate multiple environmental factors—including water quality, habitat structure, and food availability—their population responses provide a reliable signal of ecosystem condition under different farming regimes.

Although no biologically meaningful difference was detected in May (Cohen’s *d* ≈ 0), a marked and sustained divergence emerged from June onwards, with effect sizes consistently exceeding *d* = 1.0 across all abundance and biomass metrics throughout the remainder of the cultivation period (June–September; Table 2, Table 3 and Table S3). This temporal pattern suggests that the ecological benefits of eco-friendly management accumulate progressively over the growing season, rather than manifesting as an immediate response to differences in farming inputs, and that these differences reflect persistent environmental conditions rather than short-term fluctuations. Similar effects of agricultural management on aquatic invertebrate communities have been reported from rice-growing regions elsewhere [19], suggesting that farming practices broadly influence aquatic biodiversity across paddy ecosystems.

### 4.2. Environmental and Biological Drivers of Community Shifts

The contrasting responses of *C. auratus*, *M. anguillicaudatus*, and *O. latipes* illustrate how species with divergent body sizes and life-history strategies respond to farming practices. Large-bodied species tend to be more sensitive to environmental degradation and may have experienced more favorable environmental conditions often associated with eco-friendly management, although habitat structure was not quantitatively measured in the present study. In contrast, smaller, short-lived species are often more tolerant of anthropogenic disturbances and can persist—or even thrive—under the simplified conditions typical of conventional paddies. These body-size- and life-history-dependent responses are consistent with established ecological theory, which suggests that larger-bodied fishes generally require more stable and complex habitats, while smaller, short-lived species can exploit disturbed, resource-poor, and frequently drained, hydrologically fluctuating habitats such as managed paddies [43].

Several interactive mechanisms likely underpin these observed community shifts. First, reduced pesticide exposure may represent one plausible mechanism contributing to the lower abundance of some species in conventional systems [11], although pesticide residues were not directly measured in this study. The dramatic 10.8-fold increase in *C. auratus* abundance in eco-friendly paddies (CPUE_ind.: 2.38 ± 6.30 vs. 0.22 ± 1.12 individuals trap^−1^, *p* = 0.004; Table S1) is consistent with this species’ documented sensitivity to synthetic pesticides, particularly neonicotinoids, which can induce physiological disorders, behavioral impairments, and increased mortality in fish [44,45]. The reduction or exclusion of such chemicals under eco-friendly management may lessen this direct toxicity pathway and could facilitate the recovery of sensitive taxa, although this mechanism was not directly tested in the present study.

In addition to chemical safety, enhanced habitat quality and food resource availability further distinguish eco-friendly paddies. The application of organic fertilizers has been shown to boost benthic invertebrate communities, which serve as essential prey for various fish species [22,24]. Reduced nutrient loading from the avoidance of synthetic fertilizers also helps maintain stable water quality and prevents eutrophication [46], creating a more heterogeneous and hospitable environment. These conditions may also explain the observed shifts in community structure through competitive release. While large-bodied species thrive under chemical safety and habitat complexity, small, rapidly reproducing species like medaka (*O. latipes*) showed a numerical tendency toward conventional paddies (CPUE_ind.: 0.11 ± 0.65 vs. 0.38 ± 1.70 individuals trap^−1^, *p* = 0.702; Table S1). This difference was not statistically significant and should therefore be interpreted cautiously, although the observed numerical tendency is consistent with previous reports that this species can persist under relatively disturbed conditions.

Finally, these community-level changes have significant implications for higher trophic levels within the rice paddy food web. The higher fish density (2.3-fold higher CPUE_ind) and larger biomass (2.6-fold higher CPUE_bio_wet) observed in eco-friendly systems directly enhance foraging quality for avian predators. For instance, in the same study area, eco-friendly farming has been demonstrated to provide larger loaches (*M. anguillicaudatus*), which reduces hunting effort and increases net energy gain for great egrets (*Ardea alba*), potentially boosting their reproductive success [25].

### 4.3. Temporal Dynamics and Diversity Index Sensitivity

The monthly diversity analysis revealed that both farming systems followed similar seasonal trajectories, with diversity indices increasing gradually from May to September. However, a temporal shift was observed: in early months (May–June), conventional paddies showed slightly higher or comparable H’ and S values, whereas eco-friendly paddies tended toward higher values from July onwards. Despite this trend, no significant differences were detected in any month or index (*p* > 0.05; Table 4 and Table S2). Similar limitations of diversity metrics have been documented in other aquatic bioindicator groups: in a trait-based assessment of benthic macroinvertebrate communities in Korean rice paddies, conventional paddies showed higher species diversity and evenness than organic paddies despite lower ecosystem health scores, while functional diversity indices remained similar between farming systems, demonstrating that traditional diversity metrics can fail to capture—or even misrepresent—the ecological status of rice paddy wetlands [47]. Comparable temporal patterns have been reported in Australian rice fields, where morphospecies richness and Shannon diversity differed significantly between organic and conventional management regimes early in the growing season but converged as cultivation progressed, whereas community composition remained distinct throughout [19]. These patterns collectively suggest that community assembly in rice paddies is primarily governed by seasonal hydrological cycles acting as a dominant ecological filter, determining which species from the regional pool can colonize the fields, while farming practices strongly influence the survival and population density of those species.

This phenomenon is further explained by the constrained regional species pool typical of Korean rice paddies. The modest increase in species richness (S)—from approximately 2 species in May to 3–4 species by September, with eco-friendly paddies showing slightly higher richness than conventional ones in later months (Table S2)—reflects the cumulative colonization of species from adjacent water sources through shared irrigation canals, rather than improved habitat conditions per se. As the paddies remain inundated from spring transplanting until pre-harvest drainage in autumn, species present in the local water supply network gradually accumulate over the growing season. This is consistent with previous findings demonstrating that the type and connectivity of local water sources—such as adjacent reservoirs and irrigation canals—are primary determinants of fish assemblage composition in rice paddies [16,17], suggesting that the regional aquatic species pool, rather than farming practice, governs the initial species composition of paddy fish communities. This pattern is further supported by findings from Japanese rice paddies, where the abundance of cobitid loaches was more strongly associated with specific hydrological management practices—particularly the absence of crop rotation and earlier flood-irrigation dates—than with the farming system category itself [26], suggesting that water management conditions governing fish access and overwintering survival are more proximate determinants of fish population size than farming system classification per se. Our results therefore indicate that farming practices primarily influence the population sizes of these established species rather than facilitating the colonization of new taxa.

Population abundance often responds more rapidly than species richness because demographic processes such as survival, recruitment, and reproductive success can change within a single growing season, whereas colonization by additional species depends on dispersal, regional species pools, and habitat connectivity. This pattern is consistent with ecological theory that demographic responses generally precede changes in community richness [12,18]. In species-poor rice paddy ecosystems, these regional constraints may limit changes in richness even when local environmental quality improves. Consequently, abundance-based metrics may provide earlier and more sensitive indications of ecological recovery than traditional diversity indices.

To evaluate the relative sensitivity of these monitoring metrics, we compared the magnitude of change in fish abundance against various community-level indices. While all traditional diversity metrics—including Shannon’s (H’), Pielou’s (J’), and Simpson’s (D)—failed to show significant differences between the two farming systems (*p* > 0.05; Table 4 and Table S2), the contrast was most evident when comparing abundance with species richness (S). Specifically, the abundance ratios exhibited a substantial and statistically significant increase from June to September in eco-friendly paddies (2- to 3.6-fold; *p* < 0.001; Table 2), whereas the differences in S remained statistically non-significant (*p* = 0.206; Table 4), ranging from 0.8 to 1.7-fold with no consistent directional pattern. This contrast indicates that in such simplified aquatic ecosystems, population-level responses can be more reliable than community-level metrics for detecting environmental shifts. Monitoring programs in agricultural wetlands should therefore include abundance-based metrics alongside traditional diversity indices, particularly where a few dominant taxa limit the sensitivity of richness-based measures. More broadly, recent ecological work increasingly recommends complementing α-diversity with taxonomic, functional, and phylogenetic diversity, a valuable direction for future biomonitoring in such systems.

Beyond these univariate patterns, the multivariate analyses reinforced that management effects were expressed through community structure rather than α-diversity. Although diversity indices were statistically indistinguishable, event-level assemblage composition differed significantly between systems (PERMANOVA, *p* < 0.001), and conventional paddies exhibited greater among-event heterogeneity (PERMDISP; mean distance to centroid 0.52 vs. 0.40). This higher dispersion suggests that conventional management produces more stochastic, less predictable assemblages—potentially reflecting greater environmental variability among fields rather than a single specific disturbance mechanism—whereas eco-friendly paddies converged on a more consistent community state. Indicator species analysis further identified *C. auratus* as the sole significant indicator of eco-friendly paddies (IndVal = 0.65), corroborating its role as the taxon most responsive to management and reinforcing that compositional shifts, though invisible to diversity indices, carry a clear and interpretable ecological signal (Figure 4).

### 4.4. Regional and Global Context

The findings of this study are broadly consistent with patterns reported from other rice-growing regions of East Asia. A large-scale field study across Japanese rice paddies demonstrated that organic farming enhanced the richness and abundance of multiple taxonomic groups—including plants, spiders, dragonflies, and frogs—relative to conventional farming, confirming that eco-friendly management broadly promotes biodiversity in Asian rice agroecosystems [26]. Research conducted in Taiwanese rice fields similarly reported significantly higher macroinvertebrate abundance in organic paddies during the cultivation period compared with conventional systems [20,21,27]. However, fish assemblages in the Taiwanese study did not differ significantly between farming systems, in contrast to the pronounced differences observed in the present study. This discrepancy may reflect differences in the scale and duration of eco-friendly management between the two studies, as well as differences in the regional species pool and connectivity of irrigation networks. The present study monitored fish communities across a full growing season using standardized CPUE-based sampling, which may have provided greater statistical power to detect population-level responses than the shorter sampling periods employed in some previous studies.

At the global scale, meta-analyses of organic farming effects have consistently demonstrated that biodiversity recovery in managed agricultural landscapes occurs primarily through increases in organism abundance rather than large shifts in species composition. Bengtsson et al. [18] reported that organic farming increased species richness by approximately 30% on average, whereas organism abundance increased by nearly 50%—a pattern that closely mirrors the contrasting responses of abundance and species richness observed in the present study. An updated hierarchical meta-analysis further confirmed that organic farming increases species richness by approximately 34% on average across multiple taxonomic groups, with larger effects observed in intensively managed agricultural landscapes [12]. These findings suggest that the quantitative population increases documented in the present study are part of a broader and consistent pattern of biodiversity recovery under reduced-input farming systems, and that abundance-based metrics are likely to provide a more sensitive signal of ecological improvement than species richness alone across a wide range of agricultural systems.

Collectively, these regional and global comparisons confirm that the ecological responses documented in the present study—characterized by substantial increases in fish abundance and biomass under eco-friendly farming, without corresponding changes in species richness—are not idiosyncratic to Korean rice paddies but reflect a broader pattern of abundance-driven biodiversity recovery in simplified agricultural wetlands. These findings reinforce the value of abundance-based fish metrics as ecologically meaningful and practically applicable indicators for biomonitoring programs in rice agroecosystems across East Asia.

### 4.5. Management Implications and Ecosystem Services

Building on the regional and global comparisons presented above, the abundance-based analytical framework developed in this study provides a reliable basis for evaluating the ecological effectiveness of eco-friendly agricultural policies. Although species richness remained relatively stable due to regional biogeographic constraints, the 2.3-fold increase in CPUE_ind and 2.6-fold increase in biomass under eco-friendly farming provide clear biological evidence of improved ecological conditions at the field scale. These population-level metrics offer a quantitative and scientifically robust basis for policymakers to justify the continued support and potential expansion of eco-friendly farming subsidy programs.

Beyond field-scale responses, these differences have broader implications for ecosystem functioning in agricultural wetlands. Fish and other aquatic organisms in rice paddy ecosystems contribute to fundamental ecological processes, including nutrient cycling and organic matter processing [8], as well as prey provisioning for higher trophic organisms such as waterbirds [6]. The greater fish abundance observed in eco-friendly paddies therefore suggests not only improved habitat conditions but also enhanced ecosystem functioning across multiple trophic pathways within these agroecosystems.

In addition, increased fish availability generates cascading benefits for higher trophic organisms within the agricultural food web. Choi et al. [25] demonstrated that great egrets (*Ardea alba*) foraging in eco-friendly rice fields exhibited fewer hunting attempts and less active movement compared with those in conventional fields, attributable to the higher availability of larger-bodied loaches in eco-friendly paddies. Although overall feeding efficiency did not differ significantly between field types, eco-friendly paddies enabled egrets to acquire larger, more energy-rich prey with lower foraging costs, suggesting that the benefits of eco-friendly farming extend beyond fish assemblages to support broader food web dynamics and avian predator fitness.

Collectively, these findings indicate that eco-friendly rice farming enhances aquatic organism abundance while simultaneously supporting broader ecosystem processes and trophic interactions. Monitoring frameworks that prioritize abundance-based indicators of key taxa are therefore likely to provide a more sensitive and policy-relevant approach for evaluating the ecological outcomes of sustainable agriculture programs than frameworks relying solely on traditional diversity indices [13,14,15].

### 4.6. Study Limitations and Future Directions

This study provides a valuable field-scale assessment of fish community responses to eco-friendly and conventional farming practices across a full growing season. However, several limitations should be acknowledged. In addition, because this was an observational field study rather than a controlled experiment, causal relationships between specific farming practices and fish community responses cannot be established directly. As a single-year study, it cannot fully address long-term ecological dynamics or interannual variability in fish community responses. Multi-year monitoring is needed to confirm whether the abundance differences observed here represent stable, sustained ecological improvements under eco-friendly management, and to strengthen the evidence base for using fish communities as reliable ecological indicators in agricultural landscapes. In particular, the recorded assemblage was dominated by pollution-tolerant generalist species with broad adaptive capacity; together with the limited temporal and spatial extent, this constrains interpretation of the management effect and its generalization.

The spatial scope of this study was also limited to a single rice-farming region in Korea. Expanding comparable research to diverse geographical and climatic contexts across East Asia would enhance the generalizability of the findings and help determine whether the abundance-based monitoring framework developed here is broadly applicable across different regional species pools and irrigation systems.

Regarding sampling methodology, fyke net sampling effectively captures the small-bodied fish assemblages characteristic of Korean rice paddies. However, this method may inherently underestimate larger or more mobile species. Future studies incorporating complementary sampling approaches, including non-invasive environmental DNA (eDNA) metabarcoding, could provide a more comprehensive assessment of fish assemblages by improving the detection of rare or elusive species. Recent work has also demonstrated that standardized eDNA sampling protocols can effectively complement conventional field surveys in rice paddies, thereby enhancing biodiversity monitoring [30].

Finally, while this study documents clear differences in fish abundance and biomass between farming systems, the underlying causal mechanisms warrant further investigation. Detailed quantification of pesticide residues in paddy water, analysis of benthic invertebrate prey communities, and fine-scale assessment of habitat structure would help clarify the relative contributions of chemical, trophic, and physical pathways to the observed fish community responses. Such mechanistic investigations would further strengthen the theoretical and empirical foundation for using fish assemblages as primary indicators of agricultural ecosystem health in rice paddy landscapes.

## 5. Conclusions

This study demonstrates that abundance-based fish community metrics serve as sensitive and reliable ecological indicators of farming practice impacts in rice paddy ecosystems. Eco-friendly paddies consistently supported 2.3-fold higher fish abundance and 2.6-fold greater biomass than conventional paddies across the growing season. Although no difference was detected in May, effect sizes exceeded *d* = 1.0 across all abundance and biomass metrics from June through September, providing robust empirical evidence that eco-friendly management substantially improves aquatic ecological conditions at the field scale.

Species-specific responses further revealed the ecological mechanisms underlying these farming system effects. *C. auratus* was markedly more abundant in eco-friendly paddies, and *M. anguillicaudatus* was about 2.7-fold, indicating strong positive responses to eco-friendly farming practices for larger-bodied species sensitive to pesticide exposure and habitat degradation. In contrast, *O. latipes* showed a numerical tendency toward higher abundance in conventional paddies, though this difference was not statistically significant, consistent with this species’ known tolerance of disturbed environments.

Traditional diversity indices—including Shannon’s diversity (H’), Simpson’s dominance (D), Pielou’s evenness (J’), and species richness (S)—failed to detect significant differences between farming systems throughout the growing season. This insensitivity reflects the inherent structural constraints of simplified rice paddy fish communities, where a small number of highly dominant species inherently constrain the discriminatory power of community-level diversity metrics. In such ecologically simplified systems, abundance-based and species-specific indicators provide greater diagnostic power and ecological relevance for detecting management-driven environmental change.

Collectively, these findings indicate that in species-poor, dominance-skewed rice paddy systems, abundance-based and species-specific metrics provide a more sensitive signal of management effects than α-diversity indices, and should therefore be considered a valuable complement to, rather than a replacement for, traditional diversity-based biomonitoring. The substantial increases in fish abundance and biomass under eco-friendly farming also reflect enhanced ecosystem functioning across multiple trophic pathways within agricultural landscape. Because these inferences derive from a single growing season in one region, however, they should be regarded as specific to comparable systems and require multi-year, multi-region validation before generalization to Asian rice landscapes; within that scope, they offer a quantitative basis to help inform eco-friendly farming subsidy programs in Korea and across East Asia.

Future research should expand both temporal and spatial scales of investigation to confirm the long-term stability of abundance-based indicators and to assess their generalizability across diverse regional species pools and irrigation systems. Further mechanistic studies quantifying pesticide residues, benthic invertebrate prey communities, and fine-scale habitat structure will help clarify the causal pathways linking farming practices to fish community responses. Integrating abundance-based metrics with species-specific indicators and non-invasive approaches such as eDNA metabarcoding will provide a robust and comprehensive framework for tracking progress toward biodiversity conservation and sustainable agriculture goals across Asian rice landscapes.

## Figures and Tables

**Table 1:** Species composition and abundance of fish assemblages in eco-friendly and conventional rice paddies. Values are counts (number of individuals recorded); values in parentheses indicate the relative abundance (%) of each species within each farming system and across all samples for the Total column.

Family	Scientific Name	Common Name	Eco-Friendly (*n* = 48)	Conventional (*n* = 46)	Total
Cyprinidae	*Carassius auratus*	Goldfish	311 (16.5%)	23 (2.8%)	334 (12.4%)
	*Pseudorasbora parva*	Topmouth gudgeon	5 (0.3%)	1 (0.1%)	6 (0.2%)
	*Aphyocypris chinensis*	Chinese bleak	225 (11.9%)	201 (24.7%)	426 (15.8%)
	*Abbottina rivularis*	Chinese false gudgeon	1 (0.1%)	2 (0.2%)	3 (0.1%)
	*Zacco platypus*	Pale chub	6 (0.3%)	6 (0.7%)	12 (0.4%)
Cobitidae	*Misgurnus anguillicaudatus*	Oriental weatherloach	1298 (68.8%)	484 (59.3%)	1782 (65.9%)
	*M. mizolepis*	Chinese muddy loach	7 (0.4%)	4 (0.5%)	11 (0.4%)
Adrianichthyidae	*Oryzias latipes*	Japanese medaka	11 (0.6%)	53 (6.5%)	64 (2.4%)
Centrarchidae	*Lepomis macrochirus*	Bluegill	1 (0.1%)	4 (0.5%)	5 (0.2%)
Gobiidae	*Rhinogobius giurinus*	Barcheek goby	3 (0.2%)	3 (0.4%)	6 (0.2%)
	*Tridentiger brevispinis*	Trident goby	0 (0.0%)	5 (0.6%)	5 (0.2%)
	*T. bifasciatus*	Shokihaze goby	0 (0.0%)	2 (0.2%)	2 (0.1%)
Osphronemidae	*Macropodus ocellatus*	Roundtail paradisefish	20 (1.1%)	28 (3.4%)	48 (1.8%)
Total			1888 (100%)	816 (100%)	2704 (100%)

**Table 2:** Individual-based catch per unit effort (CPUE_ind; individuals trap^−1^) of fish in eco-friendly and conventional rice paddies. Values are mean ± SD (*n* = number of sampling events). Effect sizes are Cohen’s *d*; *p*-values are from a negative binomial generalized linear mixed model (GLMM; log link, field-level random intercept, log-trap offset), with monthly contrasts estimated as mean ± SD. ** *p* < 0.01, *** *p* < 0.001; n.s., not significant.

Sampling Period	CPUE_ind (Individuals Trap^−1^, Mean ± SD, *n*)		Cohen’s *d*	*p*-Value
	Eco-Friendly	Conventional		
May	3.29 ± 3.95, 8	4.59 ± 6.37, 9	−0.24	0.557 (n.s.)
June	12.23 ± 10.63, 10	3.41 ± 3.91, 9	1.08	0.001 **
July	10.43 ± 5.29, 10	4.13 ± 5.99, 9	1.12	0.007 **
August	13.62 ± 9.30, 10	4.50 ± 4.80, 10	1.23	0.002 **
September	28.37 ± 15.86, 10	13.98 ± 10.46, 9	1.06	0.114 (n.s.)
All months	14.02 ± 12.68, 48	6.09± 7.48, 46	0.76	<0.001 ***

**Table 3:** Wet-biomass catch per unit effort (CPUE_bio_wet; g trap^−1^) of fish in eco-friendly and conventional rice paddies. Values are mean ± SD (*n* = number of sampling events). Effect sizes are Cohen’s *d*; *p*-values are from a Tweedie GLMM (log link, field random intercept, log-trap offset) with monthly estimate marginal-means contrasts. * *p* < 0.05, ** *p* < 0.01, *** *p* < 0.001; n.s., not significant.

Sampling Period	CPUE_bio_wet (g trap^−1^, Mean ± SD, *n*)		Cohen’s *d*	*p*-Value
	Eco-Friendly	Conventional		
May	9.18 ± 10.12, 8	7.86 ± 4.44, 9	0.17	0.795 (n.s.)
June	22.04 ± 13.54, 10	4.11 ± 3.20, 9	1.78	<0.001 ***
July	12.05 ± 7.39, 10	4.31 ± 5.59, 9	1.17	0.008 **
August	21.75 ± 11.78, 10	6.11 ± 7.53, 10	1.58	<0.001 ***
September	49.23 ± 22.55, 10	22.63 ± 23.78, 9	1.15	0.016 *
All months	23.42 ± 19.74, 48	8.94 ± 13.09, 46	0.86	<0.001 ***

**Table 4:** α-diversity indices (Shannon–Wiener H’, Simpson’s D, Pielou’s evenness J’, species richness S; Mean ± SD) of fish assemblages in eco-friendly and conventional rice paddies. *p*-values are from generalized linear mixed models with a field-level random intercept (Poisson for S; Gaussian for H’, D, J’). n.s., not significant.

Index	Eco-Friendly (Mean ± SD, *n* = 48)	Conventional (Mean ± SD, *n* = 46)	*p*-Value
*H’*	0.47 ± 0.39	0.39 ± 0.43	0.358 (n.s.)
*D*	0.73 ± 0.23	0.77 ± 0.24	0.422 (n.s.)
*J’*	0.60 ± 0.20	0.66 ± 0.23	0.267 (n.s.)
*S*	2.54 ± 1.32	2.14 ± 1.46	0.206 (n.s.)

## Data Availability

The data presented in this study are available on request from the corresponding author. The raw dataset was also provided during submission.

## Supplementary Materials

The following supporting information can be downloaded at: https://www.mdpi.com/article/10.3390/biology15161411/s1, Table S1: Species-specific abundance of fish taxa occurring in ≥5% of sampling events, with log_10_[(N_EF_ + 1)/(N_CF_ + 1)] and system effects from negative binomial GLMMs (field random intercept, log-trap offset); Table S2: Monthly diversity indices for eco-friendly and conventional rice paddies from May to September; Table S3: Dry-biomass catch per unit effort of fish in eco-friendly and conventional rice paddies; Table S4. Model selection for the abundance and biomass GLMMs; Figure S1. Simulation-based residual diagnostics (DHARMa) for the negative binomial abundance GLMM; Code S1: annotated R analysis script (R 4.6.1.); Data S1: raw dataset.

## References

[1] FAO (2026). FAOSTAT Database.

[2] Bambaradeniya C.N.B., Amarasinghe F.P. (2003). Biodiversity Associated with the Rice Field Agroecosystem in Asian Countries: A Brief Review.

[3] Natuhara Y. (2013). Ecosystem services by paddy fields as substitutes of natural wetlands in Japan. Ecol. Eng..

[4] Luo Y., Fu H., Traore S. (2014). Biodiversity conservation in rice paddies in China: Toward ecological sustainability. Sustainability.

[5] Asare E., Mantyka-Pringle C., Anderson E., Belcher K., Clark R. (2022). Evaluating ecosystem services for agricultural wetlands: A systematic review and meta-analysis. Wetl. Ecol. Manag..

[6] Fernando C.H. (1993). Rice field ecology and fish culture: An overview. Hydrobiologia.

[7] Xie J., Hu L., Tang J., Wu X., Li N., Yuan Y., Yang H. (2011). Ecological mechanisms underlying the sustainability of the agricultural heritage rice–fish coculture system. Proc. Natl. Acad. Sci. USA.

[8] Ji Z.-J., Zhao L.-F., Zhang T.-J., Dai R.-X., Tang J.-J., Hu L.-L., Chen X. (2023). Coculturing rice with aquatic animals promotes ecological intensification of paddy ecosystem. J. Plant Ecol..

[9] Tilman D., Fargione J., Wolff B., D’Antonio C., Dobson A., Howarth R., Schindler D., Schlesinger W.H., Simberloff D., Swackhamer D. (2001). Forecasting agriculturally driven global environmental change. Science.

[10] Tilman D., Cassman K.G., Matson P.A., Naylor R., Polasky S. (2002). Agricultural sustainability and intensive production practices. Nature.

[11] Geiger F., Bengtsson J., Berendse F., Weisser W.W., Emmerson M., Morales M.B., Ceryngier P., Liira J., Tscharntke T., Winqvist C. (2010). Persistent negative effects of pesticides on biodiversity and biological control potential on European farmland. Basic Appl. Ecol..

[12] Tuck S.L., Winqvist C., Mota F., Ahnström J., Turnbull L.A., Bengtsson J. (2014). Land-use intensity and the effects of organic farming on biodiversity: A hierarchical meta-analysis. J. Appl. Ecol..

[13] Karr J.R. (1981). Assessment of biotic integrity using fish communities. Fisheries.

[14] Hughes R.M., Kaufmann P.R., Herlihy A.T., Kincaid T.M., Reynolds L., Larsen D.P. (1998). A process for developing and evaluating indices of fish assemblage integrity. Can. J. Fish. Aquat. Sci..

[15] Yoder C.O., Rankin E.T., Davis W.S., Simon T.P. (1995). Biological criteria program development and implementation in Ohio. Biological Assessment and Criteria: Tools for Water Resource Planning and Decision Making.

[16] Katano O., Hosoya K., Iguchi K.I., Yamaguchi M., Aonuma Y., Kitano S. (2003). Species diversity and abundance of freshwater fishes in irrigation ditches around rice fields. Environ. Biol. Fishes.

[17] Kim S., Park H., Park S. (2016). Distribution of fish and amphibian in rice fields near the Yedang Reservoir in Korea. Korean J. Environ. Ecol..

[18] Bengtsson J., Ahnström J., Weibull A.-C. (2005). The effects of organic agriculture on biodiversity and abundance: A meta-analysis. J. Appl. Ecol..

[19] Wilson A.L., Watts R.J., Stevens M.M. (2008). Effect of different management regimes on aquatic macroinvertebrate diversity in Australian rice fields. Ecol. Res..

[20] Rizo-Patrón F.V., Kumar A., McCoy Colton M.B., Springer M., Trama F.A. (2013). Macroinvertebrate communities as bioindicators of water quality in conventional and organic irrigated rice fields in Guanacaste, Costa Rica. Ecol. Indic..

[21] Melo S., Stenert C., Dalzochio M.S., Maltchik L. (2015). Development of a multimetric index based on aquatic macroinvertebrate communities to assess water quality of rice fields in Southern Brazil. Hydrobiologia.

[22] Pérez-Méndez N., Martínez-Eixarch M., Llevat R., Mateu D., Marrero H.J., Cid N., Catala-Forner M. (2023). Enhanced diversity of aquatic macroinvertebrate predators and biological pest control but reduced crop establishment in organic rice farming. Agric. Ecosyst. Environ..

[23] Gong S., Zhou X., Zhu X., Huo J., Faghihinia M., Li B., Zou Y. (2023). Organic rice cultivation enhances the diversity of above-ground arthropods but not below-ground soil eukaryotes. Agric. Ecosyst. Environ..

[24] Han M.S., Nam H.K., Kang K.K., Kim M., Na Y.E., Kim H.R., Kim M.H. (2013). Characteristics of benthic invertebrates in organic and conventional paddy field. Korean J. Environ. Agric..

[25] Choi G., Do M.S., Son S.J., Nam H.K. (2025). Feeding behavior and prey characteristics of great egrets (*Ardea alba*) in eco-friendly and conventional rice fields in South Korea. Sci. Rep..

[26] Katayama N., Osada Y., Mashiko M., Baba Y.G., Tanaka K., Kusumoto Y., Okubo S., Ikeda H., Natuhara Y. (2019). Organic farming and associated management practices benefit multiple wildlife taxa: A large-scale field study in rice paddy landscapes. J. Appl. Ecol..

[27] Song J.-S., Kuo C.-C. (2022). Farming practice affects rice field animal biota during cultivation but not fallow periods in Taiwan. Ecosphere.

[28] National Agricultural Products Quality Management Service (NAQS) (2025). Status of Eco-Friendly Agricultural Product Certification.

[29] Statistics Korea (KOSIS) (2025). Agricultural Area Survey.

[30] Yamamoto S., Katayama N., Yamasako J., Ito K., Okubo S., Baba Y.G. (2025). Optimal environmental DNA sampling strategy for comprehensively assessing biodiversity in rice paddy fields. Environ. DNA.

[31] Kim I.S. (1997). Illustrated Encyclopedia of Fauna & Flora of Korea.

[32] Kim I.S., Park J.Y. (2002). Freshwater Fishes of Korea.

[33] R Core Team (2026). R: A Language and Environment for Statistical Computing.

[34] Brooks M.E., Kristensen K., van Benthem K.J., Magnusson A., Berg C.W., Nielsen A., Skaug H.J., Mächler M., Bolker B.M. (2017). glmmTMB balances speed and flexibility among packages for zero-inflated generalized linear mixed modeling. R J..

[35] Hartig F. (2022). DHARMa: Residual Diagnostics for Hierarchical (Multi-Level/Mixed) Regression Models.

[36] Lenth R.V. (2026). emmeans: Estimated Marginal Means, Aka Least-Squares Means.

[37] Cohen J. (1988). Statistical Power Analysis for the Behavioral Sciences.

[38] Benjamini Y., Hochberg Y. (1995). Controlling the false discovery rate: A practical and powerful approach to multiple testing. J. R. Stat. Soc. Ser. B Stat. Methodol..

[39] Anderson M.J. (2001). A new method for non-parametric multivariate analysis of variance. Austral Ecol..

[40] Oksanen J., Simpson G.L., Blanchet F.G., Kindt R., Legendre P., Minchin P.R., O’Hara R.B., Solymos P., Stevens M.H.H., Szoecs E. (2025). Vegan: Community Ecology Package.

[41] De Cáceres M., Legendre P. (2009). Associations between species and groups of sites: Indices and statistical inference. Ecology.

[42] Wickham H. (2016). ggplot2: Elegant Graphics for Data Analysis.

[43] Winemiller K.O., Rose K.A. (1992). Patterns of life-history diversification in North American fishes: Implications for population regulation. Can. J. Fish. Aquat. Sci..

[44] Hayasaka D., Korenaga T., Sánchez-Bayo F., Goka K. (2012). Differences in ecological impacts of systemic insecticides with different physicochemical properties on biocenosis of experimental paddy fields. Ecotoxicology.

[45] Kakuta I., Takase K. (2024). Exposure to neonicotinoid pesticides induces physiological disorders and affects color performance and foraging behavior in goldfish. Physiol. Rep..

[46] Benton T.G., Vickery J.A., Wilson J.D. (2003). Farmland biodiversity: Is habitat heterogeneity the key?. Trends Ecol. Evol..

[47] Kim M.K., Kim D.G. (2025). Comparative analysis of functional diversity and ecosystem health in organic and conventional rice paddies: A trait-based assessment of benthic macroinvertebrate communities. Entomol. Res..

