# Supplementary material for: Fish Assemblages Distinguish Eco-Friendly from Conventional Rice Paddies Through Abundance and Biomass Rather than Diversity
Source: Biology (Basel). 2026 Aug 17;15(16):1411. doi: 10.3390/biology15161411 (PMC13509312; doi:10.3390/biology15161411)
Supplement: Supplementary file 1 [file biology-15-01411-s001.zip › Supplementary_Materials_FV.pdf]

Supplementary Materials

Fish Assemblages Distinguish Eco-Friendly from Conventional Rice Paddies through Abundance and Biomass Rather than Diversity

Byung-Mo Lee, Myung-Hyun Kim, Soon-Kun Choi, Jinu Eo, Sang-Min Jun

Table S1. Species-specific abundance ($CPUE_{ind}$; individuals $trap^{-1}$, mean ± SD) of fish taxa occurring in ≥ 5% of sampling events, with $log_{10}[(N_{EF} + 1)/(N_{CF} + 1)]$ and system effects from negative binomial GLMMs (field random intercept, log-trap offset). p-values were adjusted for multiple comparisons using the Benjamini–Hochberg false discovery rate (FDR). Positive log_{10} ratios indicate higher abundance in eco-friendly paddies. ** $p < 0.01$, *** $p < 0.001$; n.s., not significant.

Scientific name	Eco-friendly (Mean ± SD)	Conventional (Mean ± SD)	Log_{10} ratio	FDR p	Sig.
Misgurnus anguillicaudatus	9.58 ± 8.63	3.57 ± 5.50	0.43	<0.001	***
Carassius auratus	2.38 ± 6.30	0.22 ± 1.12	1.11	0.004	**
Aphyocypris chinensis	1.62 ± 2.69	1.47 ± 4.11	0.05	0.702	n.s.
Oryzias latipes	0.11 ± 0.65	0.38 ± 1.70	−0.65	0.702	n.s.
Macropodus ocellatus	0.15 ± 0.43	0.21 ± 0.64	−0.14	0.702	n.s.
Misgurnus mizolepis	0.06 ± 0.30	0.03 ± 0.12	0.20	0.702	n.s.
Rhinogobius giurinus	0.02 ± 0.10	0.03 ± 0.12	0.00	0.949	n.s.

Table S2. Monthly diversity indices (Shannon–Wiener H′, Simpson's D, Pielou's evenness J′, species richness S) for eco-friendly and conventional rice paddies from May to September. Differences between farming systems were evaluated using two-sided Mann–Whitney U tests ($\alpha = 0.05$). Sampling events with no captured individuals were excluded.

Month	Index	Eco-friendly (Mean ± SD)	Conventional (Mean ± SD)	p-value
May	H′	0.22 ± 0.31	0.35 ± 0.39	0.508
	D	0.86 ± 0.20	0.78 ± 0.24	0.508
	J′	0.76 ± 0.23	0.68 ± 0.18	0.571
	S	1.50 ± 0.76	1.89 ± 1.05	0.529
June	H′	0.25 ± 0.26	0.36 ± 0.51	0.967
	D	0.85 ± 0.16	0.79 ± 0.29	1.000
	J′	0.54 ± 0.22	0.79 ± 0.24	0.171
	S	1.70 ± 0.67	1.78 ± 1.09	0.866
July	H′	0.47 ± 0.38	0.35 ± 0.37	0.649
	D	0.75 ± 0.21	0.77 ± 0.24	0.918
	J′	0.58 ± 0.15	0.71 ± 0.33	0.106
	S	2.60 ± 1.35	1.75 ± 0.71	0.195
August	H′	0.62 ± 0.50	0.41 ± 0.48	0.377
	D	0.66 ± 0.28	0.77 ± 0.24	0.377
	J′	0.59 ± 0.24	0.68 ± 0.12	0.662
	S	3.10 ± 1.60	2.30 ± 2.11	0.137
September	H′	0.75 ± 0.21	0.49 ± 0.46	0.303
	D	0.57 ± 0.14	0.74 ± 0.25	0.227
	J′	0.61 ± 0.20	0.50 ± 0.24	0.368
	S	3.60 ± 0.70	3.00 ± 1.69	0.630

Table S3. Dry-biomass catch per unit effort ($CPUE_{bio\_dry}$; g $trap^{-1}$) of fish in eco-friendly and conventional rice paddies. Values are mean ± SD. Effect sizes are Cohen's d; p-values are from a Tweedie GLMM (log link, field random intercept, log-trap offset) with monthly estimated-marginal-means contrasts. * $p < 0.05$, *** $p < 0.001$; n.s., not significant.

Sampling period	Eco-friendly (Mean ± SD)	Conventional (Mean ± SD)	Cohen's d	p-value
May	1.42 ± 1.53	1.42 ± 0.69	0.00	0.932 (n.s.)
June	4.33 ± 2.76	0.87 ± 0.65	1.68	<0.001 ***
July	2.77 ± 1.68	1.08 ± 1.32	1.11	0.011 *
August	4.06 ± 2.22	1.20 ± 1.46	1.52	<0.001 ***
September	8.67 ± 3.41	4.21 ± 4.29	1.16	0.022 *
**All months**	**4.37 ± 3.39**	**1.74 ± 2.39**	**0.89**	**<0.001 *** **

Table S4. Model selection for the abundance and biomass GLMMs. Candidate error structures were fitted with identical fixed effects (farming system × month) and a field-level random intercept, and compared by Akaike Information Criterion (AIC). Abundance models used a log-trap offset on the raw counts; biomass models were fitted to the effort-adjusted per-trap response ($CPUE_{bio\_wet}$). ΔAIC is the difference from the best (lowest-AIC) model. For abundance, the negative binomial had the lowest AIC and adding zero-inflation or hurdle components did not improve fit ($\Delta AIC < 1$), so the more parsimonious negative binomial was retained; for biomass, Tweedie clearly outperformed the naive Gaussian ($\Delta AIC = 85.0$).

Response variable	Error structure	AIC	ΔAIC
CPUE_ind (abundance)	Poisson	1466.4	675.7
	**Negative binomial (selected)**	790.7	0.0
	Zero-inflated negative binomial	791.3	0.6
	Negative binomial hurdle	791.2	0.5
CPUE_bio_wet (biomass)	Gaussian (identity link)	761.1	85.0
	**Tweedie (log link; selected)**	676.1	0.0

Diagnostic basis for model choice. Simulation-based residual diagnostics (DHARMa) on the selected negative binomial abundance model indicated no residual overdispersion (dispersion test $p = 0.40$) and no zero inflation (zero-inflation test $p = 1.0$), so the more heavily parameterised zero-inflated and hurdle alternatives were not required; the Tweedie structure was likewise retained for biomass. See Figure S1.

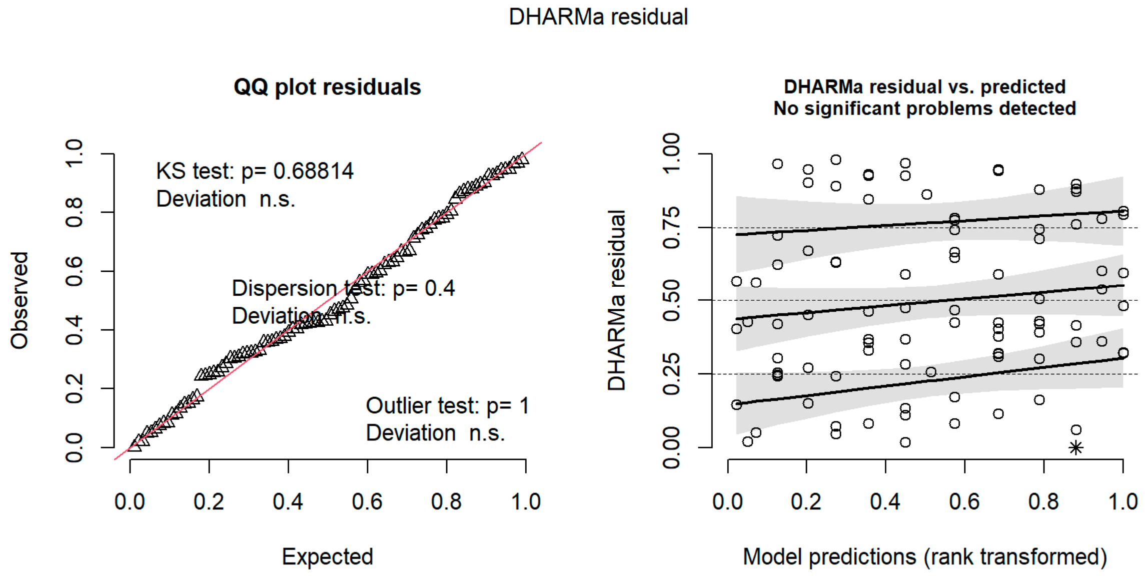

Figure S1. Simulation-based residual diagnostics (DHARMa) for the negative binomial abundance GLMM: (left) QQ plot of scaled residuals with Kolmogorov–Smirnov, dispersion, and outlier tests; (right) scaled residuals against model predictions. Uniform residuals and non-significant dispersion/zero-inflation tests support the negative binomial specification. Generated by the analysis script (FigureS1_DHARMa.png).

Data and Code

Data S1: analysis_dataset_FV.csv — the individual-level fish capture records used for all analyses (one row per captured individual; empty sampling events retained as zero-catch records).

Code S1: fish_paddy_analysis.R — a single annotated R script (R 4.6.1) that reproduces Tables 1–5 and S1–S4, Figures 1–4 and S1, and the multivariate analyses (PERMANOVA, PERMDISP, NMDS, indicator species). Package versions are recorded via sessionInfo() at the end of the script.
